# Trends of the prevalence and incidence of hypertrophic cardiomyopathy in Korea: A nationwide population-based cohort study

**DOI:** 10.1371/journal.pone.0227012

**Published:** 2020-01-13

**Authors:** Inki Moon, Seo-Young Lee, Hyung-Kwan Kim, Kyung-Do Han, Soongu Kwak, Minkwan Kim, Hyun-Jung Lee, In-Chang Hwang, Heesun Lee, Jun-Bean Park, Yeonyee E. Yoon, Yong-Jin Kim, Goo-Yeong Cho

**Affiliations:** 1 Department of Internal Medicine, Seoul National University Hospital, Seoul, Republic of Korea; 2 Department of Medical Statistics, College of Medicine, The Catholic University of Korea, Seoul, Republic of Korea; 3 Department of Cardiology, Cardiovascular Center, Seoul National University Bundang Hospital, Seongnam, Gyeonggi, South Korea; 4 Division of Cardiology, Department of Internal Medicine, Seoul National University Hospital Healthcare System Gangnam Center, Seoul, Korea; Policlinico Casilino, ITALY

## Abstract

Temporal trends of the prevalence and incidence of hypertrophic cardiomyopathy (HCM) have not been well established in Asian populations. Using the Korean National Health Insurance Services database, we identified patients with a confirmed diagnosis of HCM between 2010 and 2016. The annual prevalence and incidence of HCM, and their clinical characteristics were investigated. The prevalence of HCM has increased from 0.016% (n = 6313) in 2010 to 0.031% (n = 13,035) in 2016. During a 7-year period, 13,229 patients were newly diagnosed with HCM. The incidence rate increased from 4.15 (per 100,000 person-years) in 2010 to 5.6 in 2016. The prevalence and incidence of HCM increased with age and peaked during the 70s, with male predominance in all age groups. Chest pain is the most frequent clinical presentation followed by shortness of breath and syncope. Hypertension and dyslipidemia were the two most common comorbidities. Heart failure and atrial fibrillation was diagnosed in about 1/3 and 1/4 of patients with HCM, respectively. The prevalence and incidence of HCM gradually increased from 2010 to 2016, possibly due to heightened recognition of the disease. Given the progressively high incidence of HCM with age and high prevalence of coexisting modifiable risk factors, continued efforts are required to increase awareness regarding HCM-related symptoms and potential complications.

## Introduction

Hypertrophic cardiomyopathy (HCM) is the most common inheritable genetic cardiomyopathy and is known to be an important cause of cardiovascular morbidity and mortality across all ages [1–3]. Its prevalence in the general population has been previously estimated to be about 1:500 (0.2%) [1, 4]. However, this prevalence includes individuals with clinically unrecognized HCM in the general population by using aggressive echocardiographic screening; therefore, most of them may not seek medical attention due to the absence of cardiovascular symptoms [5]. In fact, the prevalence of about 1:500 is in stark contrast to the observation given by a more recent analysis of the U.S. claims database, where the prevalence of clinically established HCM was only approximately 1:3,000 (0.03%) [6]. Thus, the number of patients with HCM who are clinically diagnosed and seek medical care seems to be much smaller, suggesting that many individuals with HCM experience a normal life span without a need for medical attention [7]. From the viewpoint of national resource allocation, the prevalence of 0.03% is more important and more helpful for the healthcare policy establishment. Unfortunately, the prevalence and incidence of HCM in Asian populations are hitherto rarely available. Therefore, we sought to estimate the prevalence and incidence of HCM in the entire Korean population using the National Health Insurance Services (NHIS) database.

## Materials and methods

### Source of the database

This nationwide population-based cohort study used the NHIS claims database. The NHIS is a mandatory universal health insurance service for up to 97% of the entire Korean population, which holds anonymized health-related information, as previously described [8]. The remaining 3% with evidence of low income is covered by the Medical Aid Program, whose information has been incorporated into a single database since 2006. The data currently extracted from the NHIS claims database covers the entire Korean population. The NHIS claims database includes each patient’s demographics, diagnoses, healthcare utilization, and prescription data. Among all those insured by the NHIS, we collected subjects aged ≥18 years between January 1, 2010, and December 31, 2016. The study protocol conformed to the ethical guidelines of the Declaration of Helsinki and was approved by the Institutional Review Board of our institution (E-1805-051-944). As anonymized and unidentified information was applied for the analysis, informed consent was waived.

### Diagnosis of HCM

HCM was defined by 1) claims for diagnostic codes (*International Classification of Disease*, *Tenth Revision*, *Clinical Modification; ICD-10-CM*) (I42.1 or I42.2) with at least one admission or outpatient visit, and 2) registration in the *rare intractable diseases* (*RID*) program. The government-implemented *RID* program is a welfare policy extending health insurance coverage to 90% of medical costs for patients with *RID*, including HCM; therefore, *RID* registration is tightly controlled by verification with clinical and imaging evidence, physician’s certification, and independent reviews by medical experts and health insurance professionals, according to an act established by the Ministry of Health and Welfare. Also, the diagnostic accuracy of HCM using the ICM-10-CM and *RID* codes was previously validated with our institutional database, showing the accuracy of 92.6% [8].

### Clinical manifestations and comorbidities

Clinical manifestations and comorbidities were defined using the ICD-10-CM codes and the prescription lists in the NHIS database. The detailed definition of each comorbidity is described in S1 Table. These definitions were previously validated [8–10].

### Statistical analyses

Categorical variables (frequencies and percentages) were compared using the χ^2^ test or Fisher’s exact test, and continuous variables (mean ± standard deviation) were analyzed using the Student’s *t-*test or Wilcoxon-signed rank sum test for independent samples. The annual prevalence and incidence of HCM were estimated based on the cross-sectional data. The prevalence of HCM was annually calculated as the number of patients diagnosed with HCM divided by the number of total populations in the given year. The incidence of HCM was estimated annually as the number of newly registered patients with HCM divided by the number of entire populations in the given year. To strictly define incident cases of HCM, a 1-year blanking period was adopted to exclude patients who had been diagnosed before they were registered in the *RID* program. The overall and annual incident rates were presented as per 100,000 person-years. Two-sided p <0.05 was considered statistically significant. All statistical analyses were conducted with SAS version 9.3 (SAS Institute, Cary, NC, USA) and GraphPad Prism 7 software (Graph-Pad Software, La Jolla, California).

## Results

### Annual prevalence and clinical characteristics of HCM

**Fig 1:**
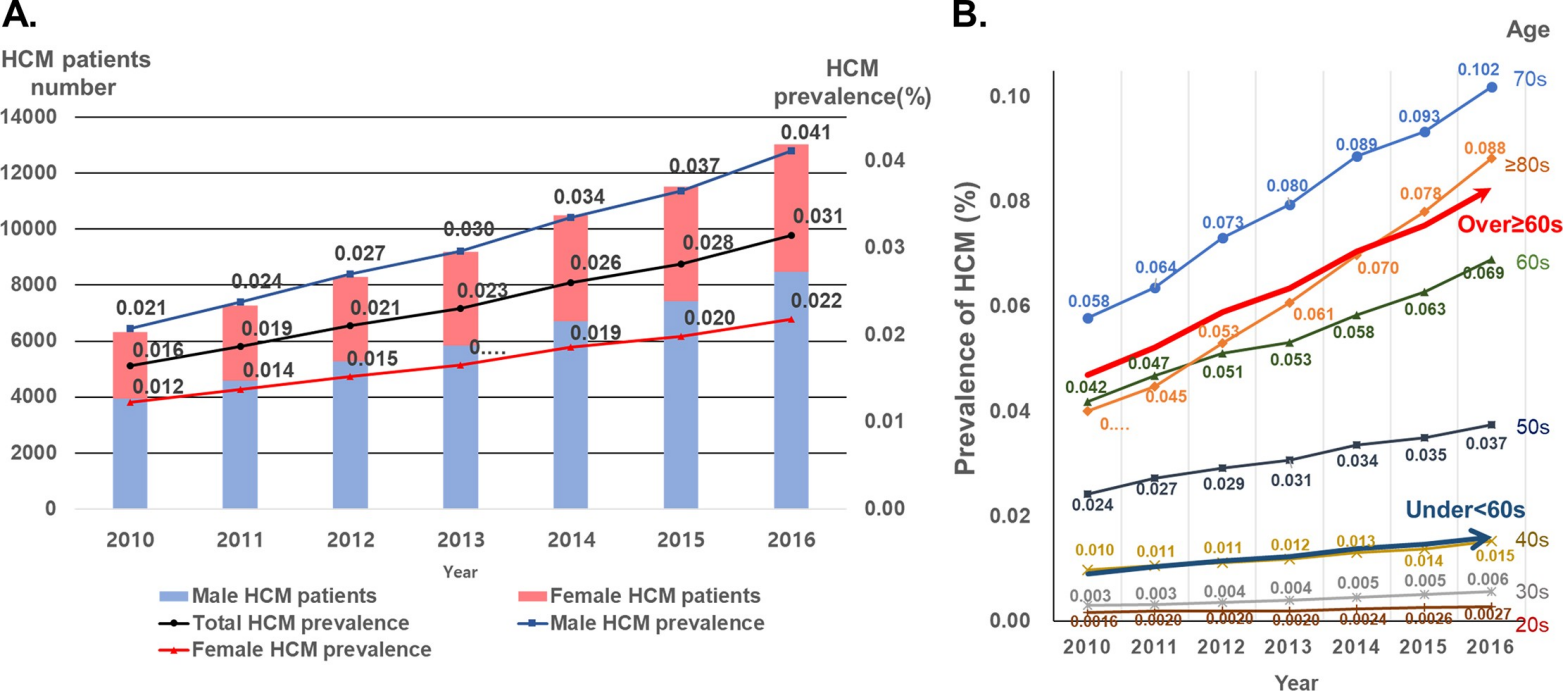
The prevalence of HCM from 2010 to 2016. ***A*.** Temporal trend of the prevalence of HCM stratified by gender. ***B*.** Age-stratified temporal trend of HCM prevalence. HCM = hypertrophic cardiomyopathy.

In all, the prevalence of HCM steadily increased by 1.9-fold from 0.016% (n = 6313) in 2010 to 0.031% (n = 13,035) in 2016. A gradual increase in the prevalence of HCM was consistently observed in both genders, with male predominance during the entire study period. The ratio of males to females was approximately 2:1 with no changes during the study period (Fig 1A, Table 1).

**Table 1:** Annular prevalence and clinical characteristics of patients with hypertrophic cardiomyopathy.

	Year						
	2010	2011	2012	2013	2014	2015	2016
Total population, N	38,427,247	38,877,086	39,416,713	39,905,161	40,430,523	40,987,643	41,498,830
HCM patients, N	6,313	7,269	8,293	9,182	10,507	11,532	13,035
Prevalence (%)	0.016	0.019	0.021	0.023	0.026	0.028	0.031
*Demographics*							
Male	3,940 (62.4)	4,577 (63.0)	5,266 (63.5)	5,844 (63.7)	6,708 (63.8)	7,427 (64.4)	8,468 (65.0)
Age (years)	60.7±12.9	60.9±13	61.5±13	61.9±13.1	62.2±13.2	62.5±13.2	62.8±13.2
Male (years)	57.6±12.1	57.9±12.2	58.4±12.2	58.9±12.3	59.2±12.3	59.7±12.4	60±12.5
Female (years)	65.7±12.8	66.1±12.7	66.8±12.6	67.2±12.7	67.4±13	67.6±13.1	68±12.8
*Clinical presentation*							
Chest pain	2,550 (40.4)	2,724 (37.5)	3,291 (40.0)	3,819 (41.6)	4,685 (44.6)	5,131 (44.5)	5,763 (44.2)
Dyspnea	552 (8.7)	572 (7.9)	684 (8.3)	827 (9.0)	965 (9.2)	1177 (10.2)	1313 (10.1)
Syncope	NA	150 (2.1)	180 (2.2)	226 (2.5)	268 (2.6)	315 (2.7)	361 (2.8)
*Comorbid diagnosis*							
Hypertension	4,299 (68.1)	4,528 (62.3)	5,692 (68.6)	6,324 (68.9)	7,064 (67.2)	7,616 (66.0)	8,310 (63.8)
Diabetes mellitus	943 (15.0)	1,169 (16.1)	1,374 (16.6)	1,597 (17.4)	1,878 (17.9)	2,109 (18.3)	2,351 (18.0)
Dyslipidemia	2,593 (41.1)	3,089 (42.5)	3,681 (44.4)	4,204 (45.8)	5,012 (47.7)	5,701 (49.4)	6,156 (47.2)
Heart failure	967 (15.3)	1,263 (17.4)	1,789 (21.6)	2,380 (25.9)	3,263 (31.1)	3,942 (34.2)	5,013 (38.5)
AF	NA	1,473 (20.3)	1,797 (21.7)	2,091 (22.8)	2,377 (22.6)	2,750 (23.9)	3,281 (25.2)
VT	667 (10.6)	740 (10.2)	867 (10.5)	996 (10.9)	1,298 (12.4)	1,512 (13.1)	1,726 (13.2)
VF	23(0.36)	32(0.44)	43(0.52)	47(0.51)	64(0.61)	74(0.64)	98(0.75)
Other cardiac arrhythmias^a^	667(10.57)	740(10.18)	867(10.45)	996(10.85)	1298(12.35)	1512(13.11)	1726(13.24)
SCD	7 (0.11)	11 (0.15)	13 (0.16)	11 (0.12)	9 (0.09)	16 (0.14)	21 (0.16)

Values presents as N (%) and mean ± SD.

^a^ Other cardiac arrhythmias include atrial / junctional / ventricular premature complexes, unspecified premature complexes, and sick sinus syndrome.

NA; not available. HCM = hypertrophic cardiomyopathy; AF = atrial fibrillation; VT = ventricular tachycardia; VF = ventricular fibrillation; SCD = sudden cardiac death.

The prevalence of HCM increased very slowly in patients aged between 20 and 59 years, whereas the prevalence sharply increased in patients aged over 60 years (Fig 1B). HCM prevalence was highest in patients aged between 70 and 79 years (Fig 1B and S2 Table). In all age categories except the age category of ≥80 years, male HCM had a numerically higher prevalence than female HCM with a male to female ratio of 2–4:1 (S1 Fig and S2 Table). HCM prevalence was similar in the age category of ≥80 years (S2 Table).

The mean age of patients with HCM remained relatively stable from 60.7±12.9 years in 2010 to 62.8±13.2 in 2016. Of note, female patients with HCM were older by approximately 8 years than their male counterparts throughout the study period (Table 1). In 2016, the most common clinical presentation was chest pain (n = 5763 [44.2%]), followed by dyspnea (n = 1313 [10.1%]). Only 361 patients (2.8%) presented with syncope in 2016. Clinical presentation with dyspnea showed an increasing trend from 8.7% in 2010 to 10.1% in 2016. Although the absolute number of cases presenting with syncope was small, its incidence tended to steadily rise. The prevalence of chest pain also increased during the study period.

Hypertension was the most common comorbidity in patients with HCM, followed by dyslipidemia. The proportion of patients with heart failure (HF) significantly increased from 15.3% in 2010 to 38.5% in 2016 (p<0.001). Atrial fibrillation, ventricular tachycardia, and ventricular fibrillation slowly increased in prevalence during the study period. Sudden cardiac death occurred in 7 patients (0.11%) in 2010 and did not change significantly during the study period (p-for-trend = 0.749).

### Incidence of HCM

**Fig 2:**
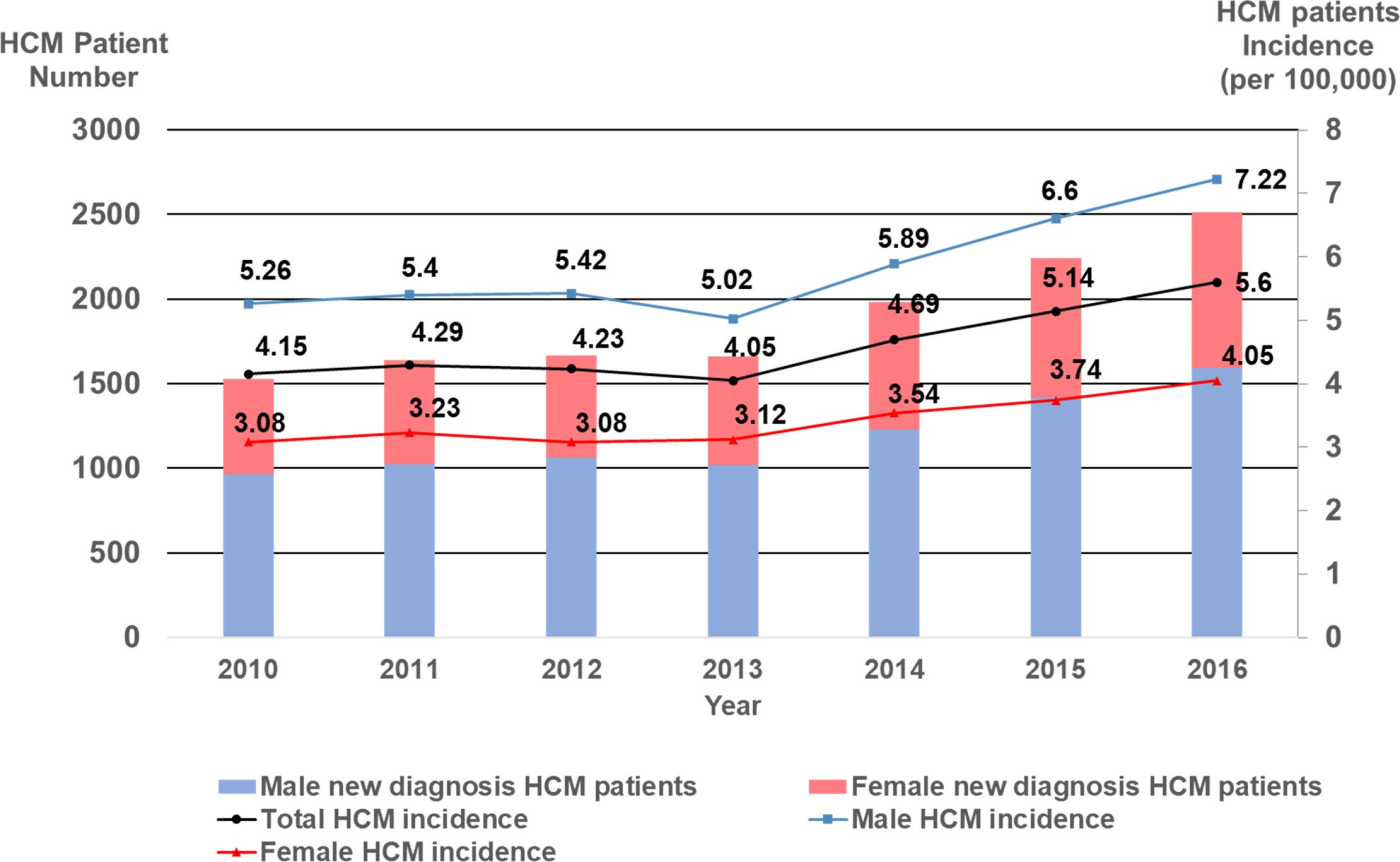
The incidence of HCM from 2010 to 2016. **Temporal trend of the incidence rate of HCM stratified by gender.** HCM = hypertrophic cardiomyopathy.

A total of 13,229 patients were newly diagnosed with HCM from 2010 to 2016. The incidence rate of HCM steadily increased from 4.15 per 100,000 person-years in 2010 to 5.6 per 100,000 person-years in 2016. The incidence rate was an approximately 1.7-fold higher in male HCM than in their female counterpart throughout the study period (Fig 2). Irrespective of gender, the incidence rate of HCM reached the highest value in the age category between 70 and 79 years, beyond which the incidence rate began to decrease (S2 Fig and S3 Table). The overall incidence of male HCM was 1.72-fold higher than that of female HCM (6.0 vs. 3.5 per 100,000 person-years, p<0.001).

**Fig 3:**
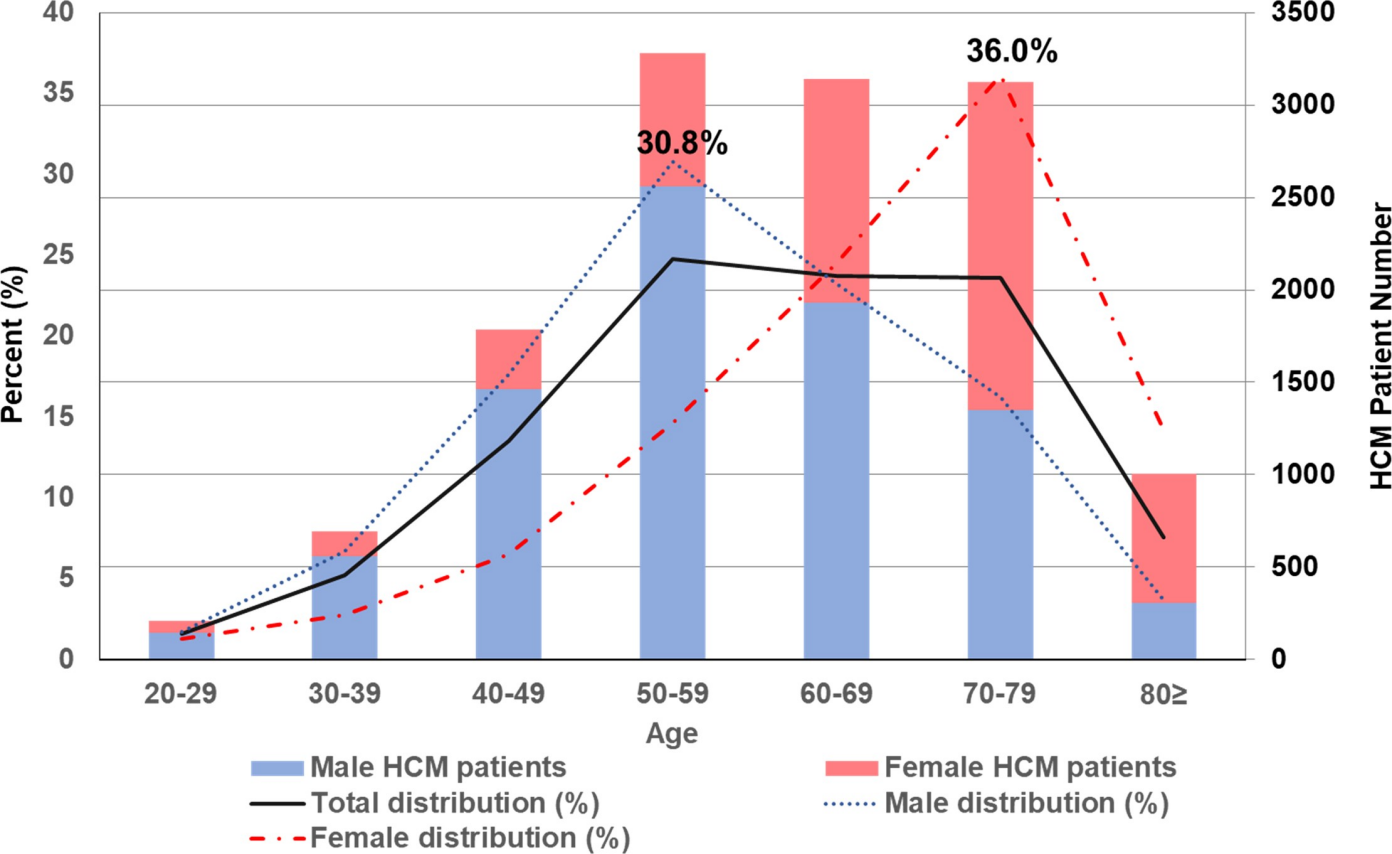
Age distribution of the newly-diagnosed HCM cohort by gender. HCM = hypertrophic cardiomyopathy.

Throughout the study period, the proportion of male HCM newly diagnosed was the highest in the age category between 50 and 59 years (30.8%), whereas its proportion in female HCM was the highest in the age category between 70 and 79 years (36.0%) (Fig 3). Thus, the peak incidence was skewed to the right in female patients with HCM with a “reversed V-shaped” curve in both genders.

## Discussion

The prevalence of HCM has been reported to be in the range of 0.02–0.23% [1]. Earlier studies from the U.S. reported that the HCM prevalence was about 0.2% (1:500 cases) [4, 11, 12]. Of note, those studies used echocardiographic screening to sensitively detect subclinical cases. Subsequent analysis using the U.S. claims database by the same research group revealed a much lower rate of clinically diagnosed HCM (0.03%) than previously thought [6]. On the other hand, the study from Germany by using the healthcare claims database reported that the prevalence of HCM was only 0.07% (1:1372) (4000 of 5,490,810 Germans) in 2015 [13]. Thus, the prevalence of HCM in the echocardiography-based population-screening studies showed a high discrepancy in that reported in the nationwide population-based studies, suggesting that HCM is still underdiagnosed and remained undetected in the general population, even in developed countries such as the U.S. and Germany. The same trend may be anticipated in Asian populations; however, there are hitherto no available studies reporting the prevalence of patients with HCM who seek medical attention. To the best of knowledge, the current study is the first to investigate the epidemiological situation of HCM in Korea based on a large nationwide NHIS claims database. We here found that the prevalence of clinically diagnosed HCM was 0.031% in 2016. Given the estimated prevalence of 1:500 or 0.2% that was carefully investigated by echocardiography-based population screening, it can be presumed that many patients with HCM are still unidentified in the general Korean population, similar to the observation from the U.S. and Germany. In fact, given the total number of Korean people, which has been estimated to be 50 million, the estimated number of cases of HCM should be 100,000. Therefore, given the total number of patients with HCM in 2016 (n = 13,035), approximately 85,000 cases of HCM are clinically unrecognized, representing the “tip-of-the-iceberg” of the disease spectrum suggested by Maron and colleagues [6]. This may be related to the lack of HCM-specific symptoms. Fortunately, the prevalence of HCM gradually increased from 6313 (0.016%) in 2010 to 13,035 (0.031%) in 2016, possibly due to the enhanced recognition of the importance and clinical implication of this disease entity, leading to more frequent screening with echocardiography. This notion is further supported by the observation that the incidence rate has progressively increased from 4.15 per 100,000 person-years in 2010 to 5.6 per 100,000 person-years in 2016 (S3 Table). To reduce the gap between the real number of cases of HCM and the number of clinically apparent patients with HCM, more efforts should be made to detect cases of subclinical HCM in the general population.

We observed in the present study that the prevalence of HCM was higher in males than in females throughout the study period. In addition, the incidence of HCM was still higher in males, with a male-to-female ratio of about 2:1. The male predominance in HCM has been previously reported [13–16]. Given the autosomal dominant inheritance, the male-to-female ratio should be 1:1. Thus, the discrepancy in the prevalence between the two genders may suggest underdiagnosis of HCM in females. We also noticed that about one-third of female HCM was first diagnosed at ages of 70–80 years, whereas about one-third of male HCM were diagnosed at ages of 50–60 years, thereby illustrating that female patients with HCM tend to have delayed diagnosis. Maron and colleagues also demonstrated that female individuals were diagnosed with HCM later in life, with more advanced disease at the initial evaluation than their male counterparts [12], leading to worse survival [17]. Given the same pattern of male predominance in the previous cohort and claims data [1, 7, 12, 13], it is likely that this observation reflects the referral bias relevant to this disease worldwide; i.e. younger and more symptomatic patients tend to be referred to the tertiary care centers for in-depth investigation [6].

In the present study, we observed that hypertension was the comorbid condition most frequently co-existed in patients with HCM between 2010 and 2016, approaching up to 70% of prevalence in patients with HCM. Hypertension has been previously reported to be present in patients with HCM, with a proportion ranging from 16.7% to 80.7% [7, 13, 17, 18]. In particular, Husser and colleagues found a high prevalence of hypertension (80.7%) in patients with HCM with a mean age of 63±17 years (S4 Table) [13]. Traditionally, co-existence of hypertension with HCM has not been naturally accepted because HCM has been regarded as a disease of the young. However, HCM is nowadays more frequently diagnosed in the elderly population. In fact, the present study observed that the prevalence of HCM was highest at ages 70–80 years for both genders, and about 1/3 of female HCM patients were newly diagnosed at ages of 70–80 years. It was perceived that the prevalence of hypertension progressively increased with age. Interestingly, hypertension was reported to be associated with delayed diagnosis of HCM [19]. Therefore, now may be the time to change the traditional concept; hypertension is a frequent comorbid condition in patients with HCM, and its presence cannot exclude the diagnosis of HCM.

In the last 20 years, many retrospective and/or observational cohort studies have found that normal life expectancy is expected in most patients with HCM, without the functional disability of disease-associated clinical events, nor the necessity for therapeutic interventions [2, 7, 20]. As supporting evidence, the average age of the first diagnosis has markedly increased more than 60 years old in recently published studies [13, 16–18]. Furthermore, a community-based cohort study without selection bias caused by selected referral to the tertiary care centers showed that a quarter of HCM patients were more than 75 years old [7]. Thus, it is not unexpected that patients with HCM can often survive to > 70 years of age, often with minimal or mild symptoms, and thus reach longevity similar to that of an age- and sex-matched general population [2]. As a result, many patients with HCM are progressively exposed to lifestyle-related disorders and die from non-HCM-related causes, including coronary artery disease [21]. In this respect, continued surveillance of modifiable risk factors such hypertension, dyslipidemia, and diabetes mellitus are of clinical implication in the contemporary HCM population.

### Limitations

Several limitations of the current study need to be acknowledged. First, the inherent limitation of the NHIS claims database did not allow for assessing clinical data such as echocardiography, electrocardiogram, or cardiac magnetic resonance imaging. Thus, information on the presence or absence of the left ventricular outflow tract obstruction could not be obtained. Second, the diagnosis of HCM and other comorbidities were based on the diagnostic codes in the NHIS claims database. Thus, the small possibility of misdiagnosis cannot be completely excluded. However, the diagnosis of HCM in the study population was verified by a meticulous review by independent medical experts according to an act on the expanding benefit coverage of the national health insurance. Besides, diagnostic validity was confirmed by our hospital data (n = 1110) with a high positive predictive value and accuracy [8]. Finally, the mean age of HCM patients in the current study was more than 60 years old, similar to the registry data from Germany [13]. The higher proportion of cardiovascular risk factors such as hypertension, hypercholesterolemia, and diabetes mellitus were observed, as well. This may be related to improved clinical care and awareness, resulting in enhanced longevity in this unique population [22]. Therefore, it is not surprising that older patients with HCM have cardiovascular risk factors more than frequently than reported previously.

## Conclusions

The prevalence of HCM progressively increased from 0.016% (n = 6313) in 2010 to 0.031% (n = 13,035) in 2016, which was in clear contrast with the prevalence of 0.2% based on a meticulous survey with echocardiography. This suggests that many patients with HCM remain undiagnosed and may be exposed to a risk for HCM-related events. Given the progressively high incidence of HCM with age and a higher rate of coexisting modifiable risk factors like hypertension, dyslipidemia, and diabetes mellitus, continued efforts are required to increase awareness regarding HCM-related symptoms and potential complications in the public and community, which can extend the opportunity for appropriate management to the HCM population that is not yet clinically identified.

## Supporting information

S1 Table Definition of each comorbidity. ICD-10-CM = the International Classification of Disease, Tenth Revision, Clinical Modification; HCM = hypertrophic cardiomyopathy; RID = rare and intractable disease; VT = ventricular tachycardia; VF = ventricular fibrillation.
(DOCX)

S2 Table Annual prevalence of HCM between 2010 and 2016 according to sex and age. Values presents as N (%). HCM = hypertrophic cardiomyopathy; IR = incidence rate.
(DOCX)

S3 Table Annual incidence of HCM between 2010 and 2016 by sex and age. Values presents as N (%). HCM = hypertrophic cardiomyopathy; IR = incidence rate.
(DOCX)

S4 Table Comorbidities, clinical presentations, and complications in patients with HCM reported previously. Values presents as mean ± standard deviation and %. HCM = hypertrophic cardiomyopathy; AF = atrial fibrillation; VT = ventricular tachycardia; VF = ventricular fibrillation; SCD = sudden cardiac death.
(DOCX)

S1 Fig Annual prevalence of HCM stratified according to age and gender. (TIF)

S2 Fig Annual incidence rate of HCM stratified according to age and gender. (TIF)
