# Supplementary material for: Trends of the prevalence and incidence of hypertrophic cardiomyopathy in Korea: A nationwide population-based cohort study
Source: PLoS One. 2020 Jan 13;15(1):e0227012. doi: 10.1371/journal.pone.0227012 (PMC6957184; doi:10.1371/journal.pone.0227012)
Supplement: S1 Table — ICD-10-CM = the International Classification of Disease, Tenth Revision, Clinical Modification; HCM = hypertrophic cardiomyopathy; RID = rare and intractable disease; VT = ventricular tachycardia; VF = ventricular fibrillation. (DOCX) [file pone.0227012.s001.docx]

**S1 Table.** **Definition of each comorbidity**

| **Diagnosis** | **ICD-10-CM code and definition** |
| --- | --- |
| HCM | I421; obstructive hypertrophic cardiomyopathy or I422; other hypertrophic cardiomyopathy (nonobstructive hypertrophic cardiomyopathy); and the RID code of V127; cardiomyopathy-related diseases |
| *Clinical manifestations*^a^ |  |
| Chest pain | I20 |
| Dyspnea | R060 |
| Syncope | R558 |
| *Comorbid diagnosis* |  |
| Hypertension^b^ | I10-I13, I15; and minimum one prescription of anti-hypertensive drug (thiazide, loop diuretics, aldosterone antagonist, alpha-/beta-blocker, calcium-channel blocker, angiotensin-converting enzyme inhibitor, and angiotensin II receptor blocker). |
| Diabetes mellitus^a^ | E11-E14; and minimum one prescription of anti-diabetic drugs (sulfonylureas, metformin, meglitinides, thiazolidinediones, dipeptidyl peptidase-4 inhibitors, α-glucosidase inhibitors, and insulin). |
| Dyslipidemia^a^ | E78 or use of lipid-lowering agents |
| Congestive heart failure^a^ | I50 |
| Atrial fibrillation^b^ | I480-484, I489 |
| VT | I47.2 |
| VF | I49.0 |
| Other cardiac arrhythmias^c^ | I49 except I49.0 |
| Sudden cardiac death | I461 |

^a^ ≥ 1 diagnosis during hospitalization or at the outpatient clinic, in the previous one year.

^b^ ≥ 1 diagnosis during hospitalization, or ≥ 2 diagnoses at the outpatient clinic, in the previous one year.

^c^ Including atrial, nodal or ventricular complexes, and sick sinus syndrome.

ICD-10-CM=the International Classification of Disease, Tenth Revision, Clinical Modification; HCM=hypertrophic cardiomyopathy; RID=rare and intractable disease; VT=ventricular tachycardia; VF=ventricular fibrillation.
