# Supplementary material for: Trends of the prevalence and incidence of hypertrophic cardiomyopathy in Korea: A nationwide population-based cohort study
Source: PLoS One. 2020 Jan 13;15(1):e0227012. doi: 10.1371/journal.pone.0227012 (PMC6957184; doi:10.1371/journal.pone.0227012)
Supplement: S2 Table — Values presents as N (%). HCM = hypertrophic cardiomyopathy; IR = incidence rate. (DOCX) [file pone.0227012.s002.docx]

**S2 Table. Annual prevalence of HCM between 2010 and 2016 according to sex and age**

|  |  | Year | | | | | | |
| --- | --- | --- | --- | --- | --- | --- | --- | --- |
|  |  | 2010 | 2011 | 2012 | 2013 | 2014 | 2015 | 2016 |
| Total population, N | | 38,427,247 | 38,877,086 | 39,416,713 | 39,905,161 | 40,430,523 | 40,987,643 | 41,498,830 |
| HCM patients, N | | 6,313 | 7,269 | 8,293 | 9,182 | 10,507 | 11,532 | 13,035 |
| Prevalence (%) | | 0.016 | 0.019 | 0.021 | 0.023 | 0.026 | 0.028 | 0.031 |
| Sex | |  |  |  |  |  |  |  |
| Male, n (%) | | 3,940 (0.021) | 4,577 (0.024) | 5,266 (0.027) | 5,844 (0.030) | 6,708 (0.034) | 7,427 (0.037) | 8,468 (0.041) |
| Female, n (%) | | 2,373 (0.012) | 2,692 (0.014) | 3,027 (0.015) | 3,338 (0.017) | 3,799 (0.019) | 4,105 (0.020) | 4,567 (0.022) |
| Age | |  |  |  |  |  |  |  |
| 20-29 |  | 117 (0.0016) | 138 (0.002) | 136 (0.002) | 136 (0.002) | 165 (0.0024) | 178 (0.0026) | 188 (0.0027) |
|  | Male, n (%) | 76 (0.002) | 95 (0.0026) | 97 (0.0027) | 93 (0.0026) | 113 (0.0032) | 119 (0.0033) | 134 (0.0036) |
|  | Female, n (%) | 41 (0.0032) | 43 (0.0013) | 39 (0.0012) | 43 (0.0013) | 52 (0.0016) | 59 (0.0018) | 54 (0.0017) |
| 30-39 |  | 256 (0.003) | 271 (0.0032) | 302 (0.0036) | 327 (0.004) | 370 (0.0046) | 404 (0.0051) | 444 (0.0057) |
|  | Male, n (%) | 202 (0.0046) | 221 (0.0051) | 240 (0.006) | 259 (0.006) | 276 (0.007) | 310 (0.008) | 349 (0.009) |
|  | Female, n (%) | 54 (0.0013) | 50 (0.0012) | 62 (0.0015) | 68 (0.0017) | 94 (0.002) | 94 (0.002) | 95 (0.002) |
| 40-49 |  | 859 (0.0098) | 930 (0.0106) | 990 (0.0112) | 1,048 (0.012) | 1,172 (0.013) | 1,229 (0.014) | 1,358 (0.015) |
|  | Male, n (%) | 672 (0.015) | 735 (0.016) | 788 (0.018) | 848 (0.019) | 948 (0.021) | 992 (0.022) | 1,110 (0.025) |
|  | Female, n (%) | 187 (0.0043) | 195 (0.0045) | 202 (0.005) | 200 (0.005) | 224 (0.005) | 237 (0.005) | 248 (0.006) |
| 50-59 |  | 1,589 (0.024) | 1,908 (0.027) | 2,182 (0.029) | 2,383 (0.031) | 2,687 (0.034) | 2,872 (0.035) | 3,120 (0.037) |
|  | Male, n (%) | 1,236 (0.038) | 1,472 (0.042) | 1,731 (0.046) | 1,875 (0.048) | 2,113 (0.053) | 2,253 (0.055) | 2,451 (0.059) |
|  | Female, n (%) | 353 (0.011) | 436 (0.012) | 451 (0,.012) | 508 (0.013) | 574 (0.014) | 619 (0.015) | 669 (0.016) |
| 60-69 |  | 1,703 (0.042) | 1,944 (0.047) | 2,137 (0.051) | 2,287 (0.053) | 2,597 (0.058) | 2,947 (0.063) | 3,497 (0.069) |
|  | Male, n (%) | 1,042 (0.054) | 1,215 (0.061) | 1,342 (0.067) | 1,492 (0.072) | 1,752 (0.082) | 2,016 (0.089) | 2,419 (0.098) |
|  | Female, n (%) | 661 (0.031) | 729 (0.034) | 795 (0.036) | 795 (0.036) | 845 (0.037) | 931 (0.039) | 1,078 (0.041) |
| 70-79 |  | 1,436 (0.058) | 1,656 (0.064) | 2,014 (0.073) | 2,346 (0.080) | 2,706 (0.089) | 2,918 (0.093) | 3,217 (0.102) |
|  | Male, n (%) | 621 (0.0622) | 719 (0.068) | 912 (0.08) | 1,085 (0.088) | 1,273 (0.099) | 1,430 (0.108) | 1,606 (0.119) |
|  | Female, n (%) | 815 (0.055) | 937 (0.061) | 1,102 (0.068) | 1,261 (0.074) | 1,433 (0.081) | 1,488 (0.083) | 1,611 (0.089) |
| ≥80 |  | 353 (0.040) | 422 (0.045) | 532 (0.053) | 655 (0.061) | 810 (0.070) | 984 (0.078) | 1,211 (0.088) |
|  | Male, n (%) | 91 (0.0358) | 120 (0.044) | 156 (0.054) | 192 (0.061) | 233 (0.068) | 307 (0.081) | 399 (0.095) |
|  | Female, n (%) | 262 (0.042) | 302 (0.045) | 376 (0.052) | 463 (0.061) | 577 (0.071) | 677 (0.077) | 812 (0.085) |

Values presents as N (%).

HCM=hypertrophic cardiomyopathy; IR=incidence rate.
