# Supplementary material for: Trends of the prevalence and incidence of hypertrophic cardiomyopathy in Korea: A nationwide population-based cohort study
Source: PLoS One. 2020 Jan 13;15(1):e0227012. doi: 10.1371/journal.pone.0227012 (PMC6957184; doi:10.1371/journal.pone.0227012)
Supplement: S3 Table — Values presents as N (%). HCM = hypertrophic cardiomyopathy; IR = incidence rate. (DOCX) [file pone.0227012.s003.docx]

**S3 Table. Annual incidence of HCM between 2010 and 2016 by sex and age**

|  |  | Year | | | | | | |
| --- | --- | --- | --- | --- | --- | --- | --- | --- |
|  |  | 2010 | 2011 | 2012 | 2013 | 2014 | 2015 | 2016 |
| Total population, N | | 38,417,525 | 38,866,147 | 39,404,486 | 39,891,685 | 40,415,826 | 40,971,303 | 41,480,795 |
| HCM patients wewly diagnosed, N | | 1,528 | 1,638 | 1,668 | 1,659 | 1,980 | 2,241 | 2,515 |
| Incidence Rate  (100,000 person-year) | | 4.15 | 4.29 | 4.23 | 4.05 | 4.69 | 5.14 | 5.6 |
| By sex, Patients (IR) | |  |  |  |  |  |  |  |
| Male | | 963 (5.26) | 1,023 (5.40) | 1,062 (5.42) | 1,016 (5.02) | 1,229 (5.89) | 1,420 (6.60) | 1,597 (7.22) |
| Female | | 565 (3.08) | 615 (3.23) | 606 (3.08) | 643 (3.12) | 751 (3.54) | 821 (3.74) | 918 (4.05) |
| By age, Patients (IR) | |  |  |  |  |  |  |  |
| 20-29 |  | 21 (0.29) | 35 (0.5) | 25 (0.36) | 24 (0.35) | 35 (0.52) | 36 (0.53) | 31 (0.45) |
|  | Male | 16 (0.43) | 29 (0.8) | 18 (0.5) | 17 (0.48) | 21 (0.59) | 21 (0.58) | 23 (0.63) |
|  | Female | 5 (0.14) | 6 (0.18) | 7 (0.21) | 7 (0.22) | 14 (0.44) | 15 (0.47) | 8 (0.25) |
| 30-39 |  | 85 (1.0) | 75 (0.89) | 80 (0.96) | 101 (1.22) | 111 (1.37) | 125 (1.57) | 116 (1.48) |
|  | Male | 68 (1.56) | 63 (1.46) | 67 (1.57) | 80 (1.9) | 83 (2.0) | 99 (2.43) | 97 (2.41) |
|  | Female | 17 (0.41) | 12 (0.29) | 13 (0.32) | 21 (0.52) | 28 (0.71) | 26 (0.67) | 19 (0.5) |
| 40-49 |  | 240 (2.73) | 227 (2.59) | 230 (2.61) | 218 (2.48) | 264 (2.97) | 274 (3.07) | 332 (3.74) |
|  | Male | 197 (4.41) | 182 (4.08) | 194 (4.32) | 186 (4.15) | 216 (4.78) | 221 (4.87) | 268 (5.95) |
|  | Female | 43 (1.0) | 45 (1.04) | 35 (0.83) | 32 (0.74) | 48 (1.09) | 53 (1.21) | 64 (1.47) |
| 50-59 |  | 375 (5.73) | 441 (6.31) | 398 (5.33) | 413 (5.33) | 494 (6.18) | 561 (6.84) | 596 (7.16) |
|  | Male | 301 (9.18) | 334 (9.54) | 332 (8.89) | 307 (7.91) | 380 (9.47) | 435 (10.56) | 471 (11.26) |
|  | Female | 74 (2.27) | 107 (3.06) | 66 (1.77) | 106 (2.74) | 114 (2.86) | 126 (3.08) | 125 (3.01) |
| 60-69 |  | 381 (9.39) | 402 (9.69) | 402 (9.62) | 359 (8.36) | 428 (9.63) | 537 (11.46) | 630 (12.45) |
|  | Male | 232 (12.06) | 244 (12.33) | 243 (12.14) | 212 (10.26) | 264 (12.31) | 336 (14.78) | 401 (16.34) |
|  | Female | 149 (6.98) | 158 (7.27) | 159 (7.30) | 147 (6.59) | 164 (7.13) | 201 (8.33) | 229 (8.79) |
| 70-79 |  | 341 (13.76) | 351 (13.51) | 416 (15.13) | 413 (14.02) | 498 (16.34) | 512 (16.41) | 593 (18.83) |
|  | Male | 130 (13.03) | 134 (12.65) | 170 (14.98) | 178 (14.44) | 226 (17.58) | 243 (18.33) | 266 (19.70) |
|  | Female | 211 (14.24) | 217 (14.11 | 246 (15.24) | 235 (13.71) | 272 (15.43) | 269 (14.99) | 237 (18.17) |
| ≥80 |  | 85 (9.68) | 107 (11.38) | 117 (11.69) | 131 (12.17) | 150 (12.96) | 196 (15.59) | 217 (15.86) |
|  | Male | 19 (7.49) | 37 (13.70) | 38 (13.19) | 36 (11.49) | 39 (11.42) | 65 (17.21) | 71 (16.95) |
|  | Female | 66 (10.57) | 70 (10.45) | 79 (11.09) | 95 (12.45) | 111 (13.61) | 131 (14.89) | 146 (15.38) |

Values presents as N (%).

HCM=hypertrophic cardiomyopathy; IR=incidence rate.
