# Supplementary material for: Trends of the prevalence and incidence of hypertrophic cardiomyopathy in Korea: A nationwide population-based cohort study
Source: PLoS One. 2020 Jan 13;15(1):e0227012. doi: 10.1371/journal.pone.0227012 (PMC6957184; doi:10.1371/journal.pone.0227012)
Supplement: S4 Table — Values presents as mean ± standard deviation and %. HCM = hypertrophic cardiomyopathy; AF = atrial fibrillation; VT = ventricular tachycardia; VF = ventricular fibrillation; SCD = sudden cardiac death. (DOCX) [file pone.0227012.s004.docx]

**S4 Table. Comorbidities, clinical presentations, and complications in patients with HCM reported previously**

| **Previous study** | Maron et al.^[7]^ | Ho et al.^[S1]^ | Cecchi et al.^[S2]^ | Olivotto et al.^[14]^ | Geske et al.^[17]^ | Ingles et al.^[18]^ | Cardim et al.^[16]^ | Husser et al.^[13]^ |
| --- | --- | --- | --- | --- | --- | --- | --- | --- |
| **Year** | 2003 | 2004 | 2005 | 2005 | 2017 | 2017 | 2018 | 2018 |
| **Country** | United States | Hong Kong | Italy | United States, Italy | United States | Australia | Portugal | Germany |
| **Study design** | Single center cohort | Single center cohort | Registry data | Multi-center cohort | Single center cohort | Single center cohort | Registry data | Claims data |
| **Number of patients** | 312 | 118 | 1677 | 969 | 3673 | 356 | 1042 | 4000 |
| **Age (years)** | - | 54 ± 18 | 44 ± 19 | 46 ± 20 | 55 ± 16 | 54 ± 16 | 53 ± 16 | 63 ± 17 |
| **Male (%)** | 55.1% | 52.5% | 62.0% | 59.0% | 55.0% | 63.5% | 59.0% | 65.0% |
| **Clinical presentations (%)** | |  |  |  |  |  |  |  |
| **Dyspnea** | 55.0% | 9.3% | 51.0% | 14.0% | 40.0% | - | 32.0% | - |
| **Chest pain** | - | 33.9% | 18.0% | 12.0% | - | - | 23.0% | - |
| **Syncope** | - | 11.9% | 12.0% | 6.0% | 15.0% | 20.8% | 9.0% | - |
| **Comorbidities (%)** |  |  |  |  |  |  |  |  |
| **Hypertension** | 16.7% | - | - | - | 46.0% | 33.2% | - | 80.7% |
| **Diabetes** | - | - | - | - | - | - | - | 26.7% |
| **Dyslipidemia** | - | - | - | - | - | - | - | 53.3% |
| **Heart failure** | - | - | - | - | - | 6.5% | - | 34.3% |
| **AF** | 18.6% | - | 18.0% | 5.0% | 18.0% | 32.5% | - | - |
| **VT** | - | - | - | - | 23.0% | 26.5% | - | - |
| **Arrhythmia** | - | - | - | - | - | - | - | 26.6% |
| **SCD** | - | - | - | 0.5% | 0.5% | 11.8% | - | - |

Values presents as mean ± standard deviation and %

HCM=hypertrophic cardiomyopathy; AF=atrial fibrillation; VT=ventricular tachycardia; VF=ventricular fibrillation; SCD=sudden cardiac death.

**Supplementary References**

[S1] Ho HH, Lee KL, Lau CP, Tse HF. Clinical characteristics of and long-term outcome in Chinese patients with hypertrophic cardiomyopathy. Am J Med. 2004;116:19-23.

[S2] Cecchi F, Olivotto I, Betocchi S, Rapezzi C, Conte MR, Sinagra G, et al. The Italian Registry for hypertrophic cardiomyopathy: a nationwide survey. Am Heart J. 2005;150:947-54.
