# Supplementary figures and images for: Trends of the prevalence and incidence of hypertrophic cardiomyopathy in Korea: A nationwide population-based cohort study
Source: PLoS One. 2020 Jan 13;15(1):e0227012. doi: 10.1371/journal.pone.0227012 (PMC6957184; doi:10.1371/journal.pone.0227012)

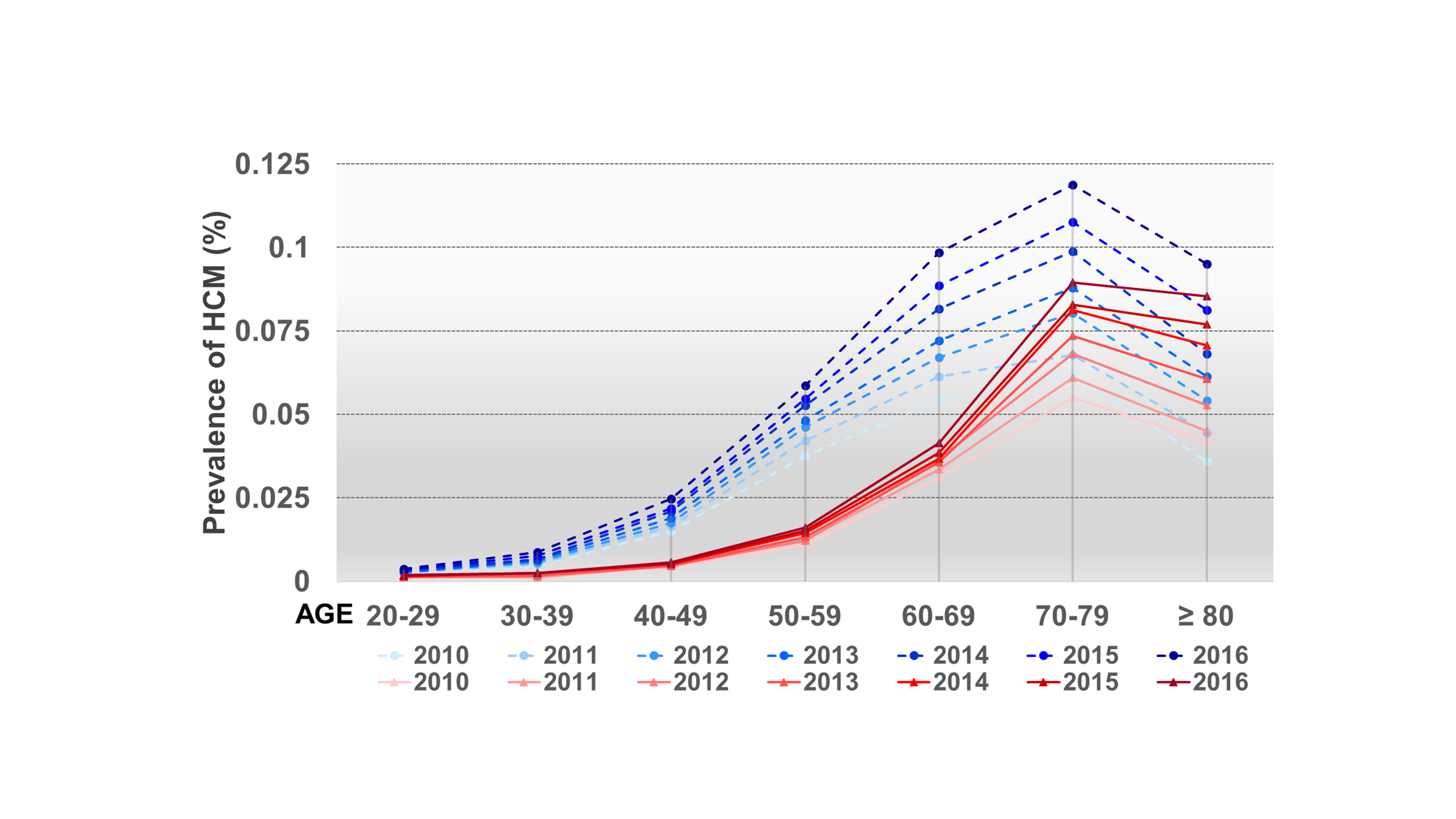

Supplement: S1 Fig — (TIF) [file pone.0227012.s005.tif]

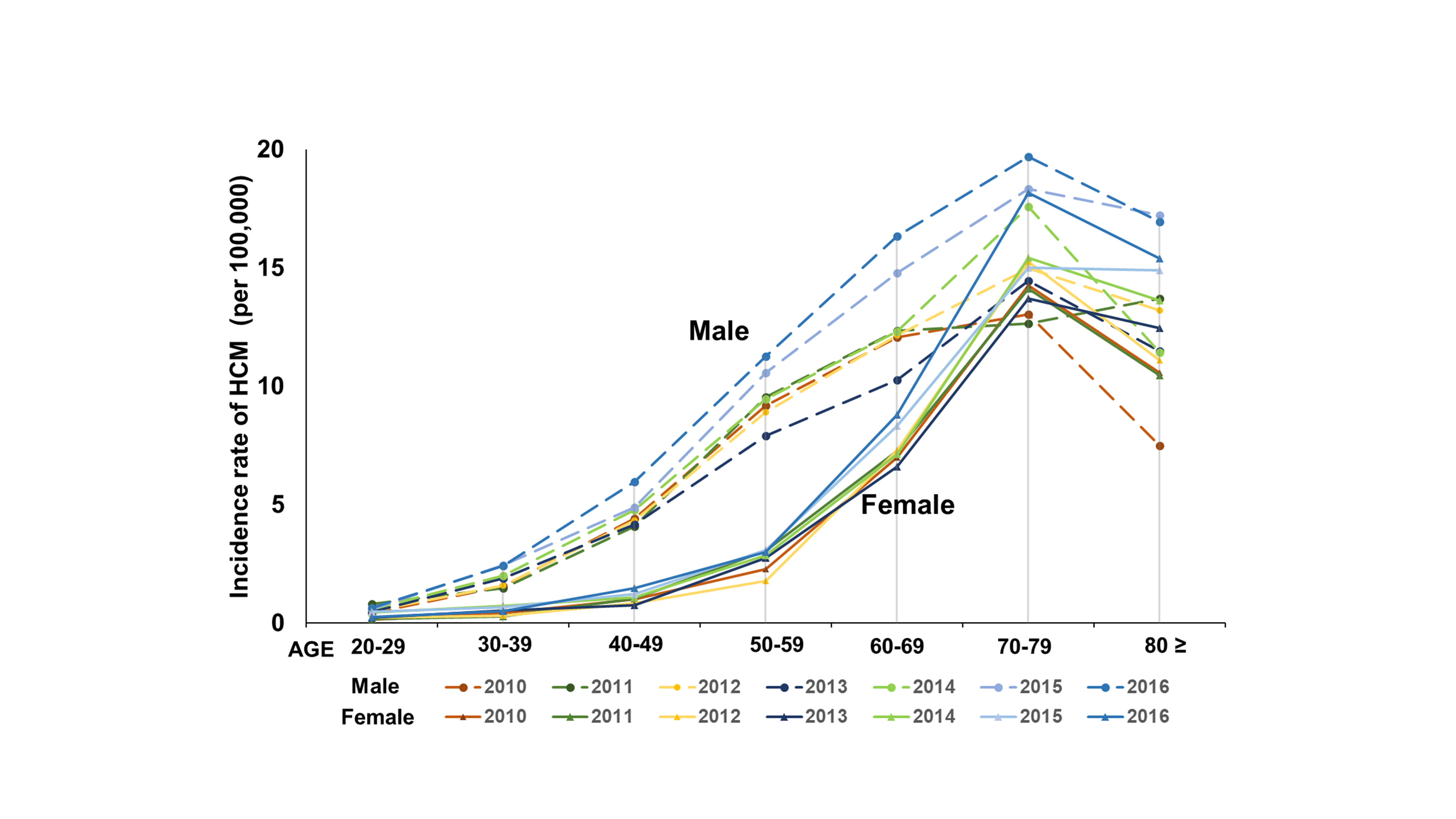

Supplement: S2 Fig — (TIF) [file pone.0227012.s006.tif]
